# Prevalence of metabolic dysfunction-associated steatotic liver disease and steatohepatitis in Türkiye: A forensic autopsy study

**DOI:** 10.1016/j.heliyon.2024.e34915

**Published:** 2024-07-20

**Authors:** Ilkay Ergenc, Erdogan Kara, Muhammed Emre Yilmaz, Coskun Ozer Demirtas, Caglayan Keklikkiran, Taner Das, Yalcin Buyuk, Cigdem Celikel, Hizir Asliyuksek, Yusuf Yilmaz

**Affiliations:** a Department of Gastroenterology, School of Medicine, Marmara University, Istanbul, Türkiye; b Institute of Liver Studies, King's College Hospital, London, UK; c Ministry of Justice, Council of Forensic Medicine, Istanbul, Türkiye; d Department of Gastroenterology, School of Medicine, Recep Tayyip Erdoğan University, Rize, Türkiye; e Institute of Legal Medicine and Forensic Sciences, Istanbul University- Cerrahpaşa, Istanbul, Türkiye; f Department of Pathology, School of Medicine, Marmara University, Istanbul, Türkiye

**Keywords:** MASLD, Steatohepatitis, Liver, Steatosis, Autopsy, Histology

## Abstract

**Background and aims:**

Metabolic dysfunction-associated steatotic liver disease (MASLD) is a growing global epidemic in Türkiye and worldwide. The aim of this study was to evaluate the prevalence and predictors of MASLD and steatohepatitis among adults who died of unnatural causes including sudden death and non-burn trauma.

**Method:**

We conducted a prospective review of all consecutive adult forensic autopsies for natural (sudden unexpected) and non-natural (Suicidal, homicidal and accidental) suspicious deaths carried out at the Istanbul Council of Forensic Medicine from February to May 2022. Four wedge biopsies were extracted from sagittal sectioned liver specimens. A liver pathologist with 20 years of experience examined each case for steatosis, ballooning, inflammation, and fibrosis.

**Results:**

Among 1797 autopsies, 62 met inclusion criteria. Overall, 43.3 % (n = 26) of autopsies showed evidence of steatotic liver disease, with a distribution of steatosis severity as: Grade I (28.3 %), Grade II (6.6 %), and Grade III (8.3 %). All these cases met at least one cardiometabolic criteria and diagnosed with MASLD. Ballooning was observed in 20.0 % of cases (5 cases grades 1 and 7 cases grade 2), and Inflammation was present in 51.7 % (9 cases with grade 0–1, 12 with 1–2, 7 with 2–3, and 3 with 5–6). Notably, 46.1 % (n = 12) of MASLD cases and 20.0 % (n = 12) of all cases were diagnosed with steatohepatitis, with three cases exhibiting delicate perisinusoidal fibrosis and one case showing portal fibrosis.

**Conclusion:**

The histopathological findings from this autopsy study confirmed the markedly high prevalence of MASLD and steatohepatitis within the general adult population, highlighting the concerning burden of steatotic liver disease in Türkiye.

## 1 Introduction

Metabolic dysfunction-associated steatotic liver disease (MASLD), previously referred as non-alcoholic fatty liver disease (NAFLD), stands as a leading cause of liver-related morbidity and mortality. Moreover, MASLD is increasingly associated with all-cause mortality and its global prevalence is around 30 % with a rising trend [1,2]. Following initial discussion and debate regarding the proposed terminology of "metabolic (dysfunction) associated fatty liver disease" (MAFLD), a multi-society Delphi consensus ultimately endorsed "MASLD" as the preferred nomenclature [[3], [4], [5]]. The new MASLD nomenclature introduces two key changes. First, it defines Steatotic Liver Disease (SLD) as an umbrella term encompassing all causes of steatosis, including alcoholic, metabolic, and their potential overlap. This broader term clarifies disease origins and avoids confusion. Second, modified diagnostic criteria prioritize cardiometabolic factors as primary drivers, requiring the presence of at least one of five specific risk factors. While the term non-alcoholic steatohepatitis (NASH) is replaced by Metabolic dysfunction-associated steatohepatitis (MASH), the focus remains on identifying and addressing the underlying metabolic triggers.

The global pandemic of MASLD shows regional variations. Latin America, North Africa, and the Middle East report the highest prevalence rates, ranging from 33.1 % to 44.4 % [6]. In Türkiye, nationwide ultrasound screenings indicate an even higher burden, with estimates ranging from 45.5 % to 60 % across different studies [[7], [8], [9]]. However, a critical knowledge gap remains regarding the precise prevalence of key drivers of disease severity and progression, namely steatohepatitis and fibrosis [10].

Ultrasound imaging offers a simple, non-invasive, and cost-effective method for community-level screening of MASLD. However, its sensitivity is notably reduced in cases of low steatosis (<20 %) and high body mass index (>40 kg/m2) [11]. Additionally, ultrasound is unable to diagnose steatohepatitis or fibrosis. Histopathology serves as the gold standard for diagnosis, but its invasive nature makes it impractical for large-scale population screening of MASLD. Autopsy series, on the other hand, offer a valuable alternative for estimating prevalence through histological assessment in targeted populations, providing crucial insights into disease burden and severity [[12], [13], [14]].

This study aims to investigate the prevalence of MASLD, MASH, and fibrosis employing the gold standard of histological analysis within a series of adult forensic autopsies.

## 2 Materials and methods

### 2.1 Study protocol

This cross-sectional study was conducted between February and May 2022 at the Mortuary Department of Council of Forensic Medicine in Istanbul. All consecutive forensic autopsies on individuals over the age of 18 years performed for natural (sudden unexpected) and non-natural (Suicidal, homicidal and accidental) suspicious deaths, were included if the death was unrelated to or caused by liver injury. We excluded those who had died more than 48 h prior to autopsy, subjects with signs of body decomposition according to the Crossley criteria, burn-related death, hospitalisation prior to death, alcohol abuse, known chronic liver disease, and medication associated with steatosis. Medical history, including smoking and alcohol habits, diabetes, hypertension, hyperlipidaemia, and cardiovascular disease, was obtained from next of kin and recorded along with age, sex, weight, height, and body mass index (BMI). Overweight was defined as BMI ≥25 kg/m^2^, and obesity was defined as BMI ≥30 kg/m^2^. The study was conducted in accordance with Helsinki Declaration. The study protocol was reviewed and approved by the Education and Scientific Research Board of the Council of Forensic Medicine (No: 21589509/2020/939).

### 2.2 Liver specimens

The same forensic medicine specialist measured the liver's volume and weight and then obtained four wedge biopsies, each measuring 2 cm × 2 cm × 1 cm, from sagittally sectioned liver specimens. Two biopsies were sampled from each lobe, one subcapsular and one intraparenchymal. All samples were preserved in 10 % buffered formaldehyde, embedded in paraffin, cut into 4 μm-thick sections, and stained with hematoxylin-eosin, silver, and Masson trichrome.

### 2.3 Histopathological examination

A liver pathologist with 20 years of experience performed all histopathological examinations. Each biopsy specimen was scored on a scale for steatosis, ballooning, inflammation, and fibrosis. Histopathologic evaluation and semiquantitative scoring were defined based on the NASH Clinical Research Network (NASH CRN) and SAF (Steatosis, activity, fibrosis) algorithm. Steatotic liver disease (SLD) was defined as macrovesicular steatosis in >5 % of hepatocytes in the absence of other liver diseases. The Non-Alcoholic Fatty Liver Disease (NAFLD) Activity Score (NAS) was calculated based on the degree of steatosis (0–3 points), lobular inflammation (0–3 points), and hepatocellular ballooning (0–2 points) and ranged from 0 to 8. Patients with at least one point of steatosis, ballooning, and lobular inflammation were diagnosed with steatohepatitis. Fig. 3 illustrates examples of histological assessment of tissues.

### 2.4 Statistical analyses

The distribution of variables was assessed using the Shapiro-Wilk test, and a p-value of less than 0.05 was considered statistically significant. Descriptive statistics were shown as median (range) for non-normally distributed variables and mean (standard deviation) for normally distributed variables. Chi-square tests were used to compare categorical variables, and t-tests or Mann-Whitney U tests were used to compare continuous variables.

## 3 Results

During the study period, 62 out of 1797 medico-legal autopsies met inclusion criteria. The median age was 51 years. 81.7 % (n = 49) was male. The median time interval between death and autopsy was 24 h. Two cases were excluded after histological examination due to moderate to severe cholestatic hepatitis suggestive of toxic or drug-induced liver injury. The reasons for autopsy were included sudden death or discovery of a dead body (45.0 %), suicide or homicide by mechanical asphyxia (hanging, strangulation and external compression) (25.0 %), blunt and sharp trauma (16.7 %), injury by firearm or sharp object (11.7 %), and death while in detention (1.7 %). The conclusive attributions for deaths were cardiovascular etiologies (33.3 %), trauma and injuries (28.3 %), hanging incidents (25.0 %), primary or metastatic brain tumours (3.4 %), intracranial haemorrhage (1.7 %), gastrointestinal haemorrhage (1.7 %), and undefined (6.7 %). The mean age was 48.5 years and the median was 51 (range: 18 to 76). Table 1 presents the clinical and demographic characteristics of the subjects.

**Table 1:** Clinical and demographic characteristics.

	No steatosis (n = 34)	MASLD (n = 26)	P-Value
Age^a^, years (Mean ± SD)	45,6 ± 16,6	52,2 ± 13,8	0.107
Sex, female	20.6 % (7)	19.2 % (5)	0.884
Height^a^, cm	169,4 ± 7,9	168,8 ± 7,9	0.781
Weight^a^, kg	70,8 ± 11,3	83,7 ± 14,2	**0.001**
BMI^a^, kg/m2	24,68 ± 3,62	29,44 ± 5,07	**0.000**
Obesity	8.8 % (3)	42.3 % (11)	**0.000**
Overweight	35.3 % (12)	46.2 % (12)	0.435
Liver weight^a^, gr	1557 ± 325	1874 ± 525	**0.017**
Reason for autopsy			0.330
Sudden unexpected death	41.2 % (14)	53.8 % (14)	
Trauma/Suicide	58.8 % (20)	46.2 % (12)	
Cause of death			0.405
Trauma/hanging	61.8 % (21)	46.2 % (12)	
Cardiovascular death	23.5 % (8)	42.3 % (11)	
Other natural etiologies	5.9 % (2)	7.7 % (2)	
Undetermined	8.8 % (3)	3.8 % (1)	
Cardiometabolic risk factors	44.1 % (15)	100.0 % (26)	**0.000**

MASLD: Metabolic-associated steatotic liver disease, BMI: Body mass index, kg: kilogram, gr: gram.

a Mean standard deviation.

A total of 68.3 % (n = 41) of all subjects exhibited cardiometabolic risk factors including obesity (21.6 %), overweight (41.6 %), diabetes mellitus (10.7 %), hypertension (28.6 %), hyperlipidaemia (17.9 %). Cardiometabolic risk factors were also identified in 51.2 % of patients died from non-cardiac reasons like trauma, or suicide. Six subjects had a history of occasional drinking at social gatherings.

**Fig. 1:**
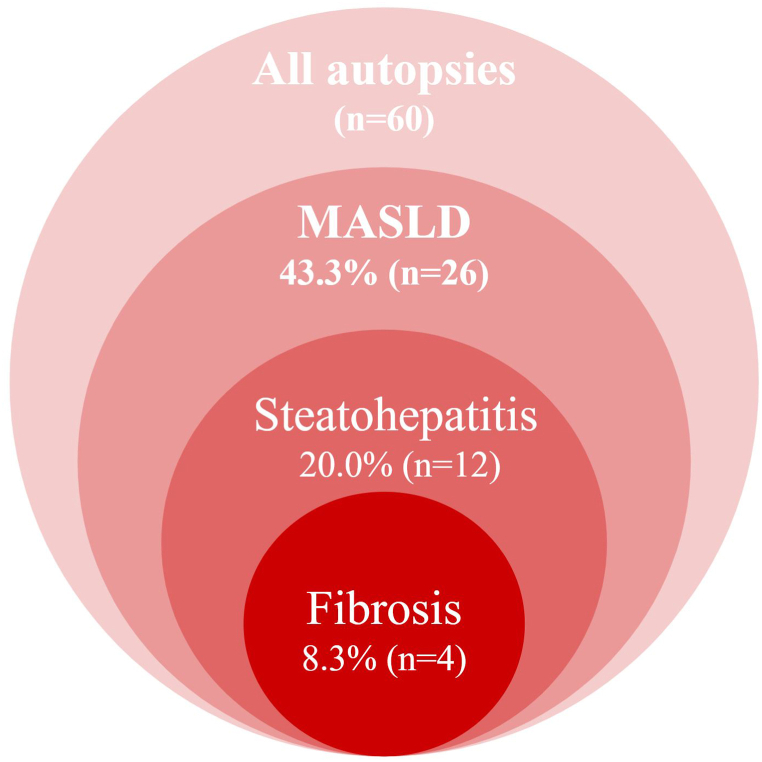
Illustrates the prevalence of metabolic-associated steatotic liver disease (MASLD), steatohepatitis, and fibrosis in the study population.

**Fig. 2:**
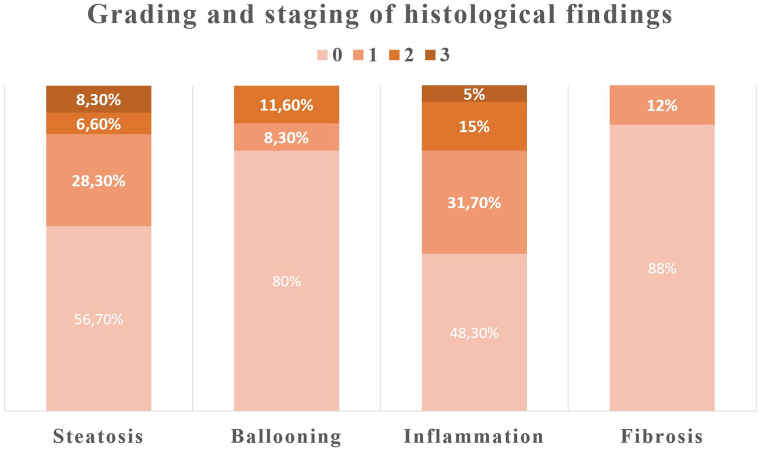
Illustrates the prevalence of different grades of steatosis, ballooning, inflammation, and fibrosis in the study population.

Overall, 43.3 % (n = 26) of autopsies showed evidence of steatotic liver disease, with a distribution of steatosis severity as: Grade I (28.3 %), Grade II (6.6 %), and Grade III (8.3 %). All patients with steatosis were having at least one cardiometabolic risk factor and diagnosed with MASLD and six of them were classified as MASLD dominant MetALD. Ballooning was observed in 20.0 % of cases (5 cases grades 1 and 7 cases grade 2), and Inflammation was present in 51.7 % (9 cases with grade 0–1, 12 with 1–2, 7 with 2–3, and 3 with 5–6). Notably, 20.0 % (n = 12) of cases were diagnosed with steatohepatitis, three of them had delicate perisinusoidal fibrosis (1a) and one had portal fibrosis (1c). Median NAFLD activity score (NAS) and steatosis SAF scores of MASLD patients were 3 (1–8) and 3 (1–7), respectively, and steatohepatitis patients were 5 (4–8) and 5 (3–7), respectively. Four subjects showed signs of hypoperfusion and apoptosis, and among the two cases without steatosis, histopathological findings indicated chronic hepatitis, with one of them presenting portal fibrosis. Fig. 1, Fig. 2 represents the histological features and final diagnosis.

**Fig. 3:**
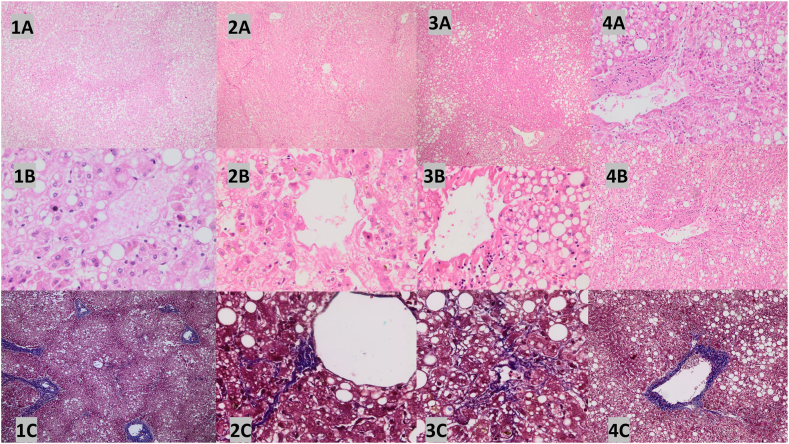
Fig. 1a: Steatosis grade 3, 1b: Mild ballooning, 1c: Portal fibrosis, 2a: Steatosis grade 1, 2b: Ballooning degeneration, 2c: Pericentral sinusoidal fibrosis, 3a: Steatosis grade 2, 3b: Mild ballooning and focal necrosis, 3c: Pericentral sinusoidal fibrosis 4a: Steatosis grade 3 and focal necrosis, 4b: Fibrosis and mononuclear cell infiltration, 4c: fibrous thickening of central vein and focal pericentral fibrosis. 1a: Steatosis involving more than 80 % of the lobule that spares only periportal hepatocytes that form the limiting plate (steatosis grade 3). 1b) Mild ballooning, but no focal necrosis/apoptosis and/or fibrosis is seen in the high-power view of the same liver section. 1c: Portal fibrosis. 2a: Pericentral steatosis involving less than 33 % of the lobule (steatosis grade 1). 2b: In the high-power view, besides ballooning degeneration there is canalicular and hepatocellular cholestasis. 2c: pericentral sinusoidal fibrosis. 3a: Pericentral steatosis involving approximately 65 % of the lobule (steatosis grade 2). 3b: In the high-power view, besides mild ballooning degeneration there is focal necrosis characterized by mononuclear cells. 3c: 3c: pericentral sinusoidal fibrosis. 4a: Pericentral steatosis involving more than 80 % of the lobule (steatosis grade 3) and many areas of focal necrosis characterized by mononuclear cells. 4b: In the high-power view of the same liver section, there is fibrosis and mononuclear cell infiltration in the wall of a central vein. 4c: fibrous thickening of central vein and focal pericentral fibrosis.

While the prevalence of steatosis was higher among individuals who died from cardiac-related causes (57.9 %) compared to those with non-cardiac death etiologies (37.2 %), this difference was not statistically significant (p = 0.130). Additionally, no significant differences were observed in NAS or SAF scores between MASLD patients who died from cardiac causes (n = 11, median NAS and SAF scores 2) and those with other etiologies (n = 15, median scores 3) (p = 0.485 and 0.410, respectively). The degrees of steatosis, ballooning, and inflammation were similar in cardiac and non-cardiac deaths. The MASLD rate was lowest among individuals under 30 years of age (3 out of 11, 27.7 %) and highest among individuals over 60 years of age (8 out of 14, 57.1 %). However, there was no significant difference in MASLD prevalence across the different age groups.

## 4 Discussion

This pioneering autopsy study in Türkiye validates the alarmingly high prevalence of MASLD in adults that was previously predicted using non-invasive methods. A previous retrospective analysis of check-up clinics in between 2007 and 2016 across Türkiye by Değertekin et al. indicated a dramatic rise in NAFLD, highlighting its prevalence among nearly half the Turkish population [7]. Expanding on previous work, Yılmaz et al. validated the high prevalence of steatotic liver disease in Türkiye through a cross-sectional ultrasound screening of a large, nationally representative cohort across eight tertiary care centres. Utilizing the MAFLD definition, they found that nearly half of the population was affected [8]. Crucially, our gold-standard histological analysis went beyond validating the high prevalence of MASLD previously seen; it also revealed a concerning burden of steatohepatitis and early-stage fibrosis within this group.

Our autopsy findings revealed an alarmingly high prevalence of steatohepatitis within the MASLD group. 46.1 % (12 out of 26) of MASLD patients exhibited steatohepatitis, representing one in five of all autopsies performed and surpassing global predictions slightly [6]. Notably, the largest published biopsy-proven cohort of Turkish patients with fatty liver disease demonstrated that over 90 % of obese individuals with liver steatosis develop steatohepatitis [15]. Therefore, the concerning high steatohepatitis rate may be a result of Türkiye's high obesity rate, which is reaching 32.2 % and increasing, the highest in Europe according to the World Health Organization [16].

Similarly, autopsy studies from other regions have reported unexpectedly high steatohepatitis prevalence. In a pediatric cohort from New York City, 40 % of NAFLD cases showed steatohepatitis [17]. Likewise, a postmortem study in Northwestern Greece found steatohepatitis in 56 % of individuals with steatosis [13]. These findings suggest that our observations in Türkiye may not be entirely unique, highlighting the potential for underestimated global burden of steatohepatitis.

Among MASLD patients with steatohepatitis in this autopsy study, 5 % showed early-stage fibrosis, suggesting a concerning quarter of the steatohepatitis cases are already on the path to advanced liver disease. In the context of steatohepatitis, mortality and morbidity risk closely correlate with histological severity, significantly increasing with worsening fibrosis [18,19]. Therefore, the high fibrosis rate in this study highlights steatotic liver disease as a major and potentially underestimated public health threat, emphasizing the necessity of predicting its burden.

One important question is to what extent can these findings be applied to the broader Turkish population. Istanbul, a bustling metropolis attracting immigrants from across Türkiye, mirrors the nation's rich ethnic composition, diverse socioeconomic conditions, and varied dietary habits and lifestyles [20]. Moreover, according to Turkish Statistical Institute (TUIK) data, 93.2 % of the population resides in urban areas [21]. Consequently, autopsies conducted at the Istanbul Council of Forensic Medicine, an official government body overseeing all medico-legal cases in the city, offer a representative sample of the entire country's population, allowing us to extrapolate our findings to Türkiye as a whole.

A striking finding of our study was the high prevalence of cardiovascular deaths among individuals with MASLD. More than 40 % of the subjects diagnosed with MASLD died due to cardiovascular reasons, while one-third of all autopsies reflected this trend. This is expected as cardiovascular death ranks as a leading cause of mortality among MASLD patients [12]. Moreover, cardiovascular diseases and MASLD share many common metabolic risk factors, making them primary risk factors for one another [22]. There is ample epidemiological data supporting the strong co-occurrence of these two conditions, as our study revealed, although causality remains unclear [23].

On the other hand, cardiovascular death was nearly twice as high in patients with MASLD compared to the other group. The high prevalence of cardiovascular deaths among MASLD patients necessitates careful interpretation of the reported prevalence rates. To minimize bias regarding cause of death, we implemented blinded assessments of medical records and employed standardized protocols for inclusion and exclusion criteria. According to Turkish Statistical Institute data, cardiovascular deaths rank first among the causes of death and accounted for 34.5 % in 2022 [24]. Therefore, our study population represents the country's average in terms of death rates and risk factors, and the results align with the previously reported co-occurrence rate of cardiovascular disease.

Our study demonstrates several notable strengths. Firstly, we employed the gold standard diagnostic method, utilizing multiple long biopsies throughout the entire liver to accurately diagnose steatosis, inflammation, and fibrosis. Secondly, we randomly selected and consecutively enrolled participants following our study design, conducting the study in Istanbul, which reflects the nation's ethnic composition and socioeconomic conditions, effectively mirroring the broader Turkish population. Thirdly, an experienced liver pathologist with over two decades of experience and a wealth of cases reviewed all biopsies blindly, ensuring objectivity and reliability of assessments. Moreover, we meticulously assessed cardiometabolic risk factors for all cases of steatosis and carefully excluded those with significant alcohol consumption or related liver disease. However, it is important to acknowledge some limitations. The relatively small study group may limit generalizability, However, given the stringent exclusion criteria we applied, we ultimately obtained a pure cohort devoid of confounding factors. Additionally, relying on family reports for alcohol history introduces potential bias. Furthermore, only one pathologist reviewed the biopsies; we did not enlist a second reader for reliability assessment. Finally, the study group consisted primarily of males. This characteristic may introduce bias, as MASLD is known to be more prevalent in men.

In conclusion, valuable histological evidence from this unique autopsy study reaffirms earlier predictions from nationwide screenings: MASLD prevalence in Türkiye is concerningly high and remain stable. More importantly, we did identify a remarkably high prevalence of steatohepatitis among MASLD subjects, along with fibrosis within the steatohepatitis population. All these findings paint a concerning picture that nearly half of the general population carries this silent threat, MASLD with marked by alarmingly high rates of steatohepatitis and fibrosis. Our results underscore the urgency of recognizing MASLD as a major public health issue in Türkiye once more. A strong interdisciplinary collaboration among hepatologists, public health authorities, and all disciplines dealing with metabolic risk factors is essential to plan preventative strategies and early interventions for safeguarding public health.

## Funding

This study has been supported by the Recep Tayyip Erdoğan University Development Foundation (Grant number: 02024007019001).

## Human ethics and consent to participate declarations

This forensic autopsy study was conducted at the National Council of Forensic Medicine with the permission of the Council's research committee. Since liver biopsy is a standard part of routine forensic autopsies in Turkey and no additional tissue samples were taken from the subjects, the institutional review board waived the requirement for informed consent, deeming the study to be of minimal risk.

## Ethics approval

The study was conducted in accordance with the revised version of Declaration of Helsinki 2008. The study protocol was approved by the Education and Scientific Research Commission of the Forensic Medicine Department, Ministry of Justice (Approval Date: September 10, 2020, Approval Number: 21589509/2020/939).

## Data availability

The data underlying this article will be shared on reasonable request to the corresponding author.

## CRediT authorship contribution statement

**Ilkay Ergenc:** Writing – review & editing, Writing – original draft, Methodology, Investigation, Formal analysis, Data curation, Conceptualization. **Erdogan Kara:** Writing – review & editing, Investigation, Data curation. **Muhammed Emre Yilmaz:** Writing – review & editing, Investigation, Data curation. **Coskun Ozer Demirtas:** Writing – review & editing, Investigation, Formal analysis, Conceptualization. **Caglayan Keklikkiran:** Writing – review & editing, Investigation, Formal analysis, Data curation, Conceptualization. **Taner Das:** Writing – review & editing, Investigation, Formal analysis, Data curation. **Yalcin Buyuk:** Writing – review & editing, Methodology, Investigation, Formal analysis, Data curation, Conceptualization. **Cigdem Celikel:** Writing – review & editing, Validation, Methodology, Investigation, Formal analysis, Data curation, Conceptualization. **Hizir Asliyuksek:** Writing – review & editing, Methodology, Investigation, Formal analysis, Conceptualization. **Yusuf Yilmaz:** Writing – review & editing, Supervision, Project administration, Methodology, Investigation, Formal analysis, Data curation, Conceptualization.

## Declaration of competing interest

The authors declare the following financial interests/personal relationships which may be considered as potential competing interests:The corresponding author, Yusuf Yilmaz, is an associate editor of this journal. If there are other authors, they declare that they have no known competing financial interests or personal relationships that could have appeared to influence the work reported in this paper.
